# ﻿Corrigendum: Salvi D, Al-Kandari M, Oliver PG, Berrilli E, Garzia M (2022) Cryptic marine diversity in the northern Arabian Gulf: an integrative approach uncovers a new species of oyster (Bivalvia: Ostreidae), *Ostreaoleomargarita*. Journal of Zoological Systematics and Evolutionary Research 2022: 7058975. https://doi.org/10.1155/2022/7058975

**DOI:** 10.3897/zookeys.1143.100087

**Published:** 2023-01-27

**Authors:** Daniele Salvi, Manal Al-Kandari, P. Graham Oliver, Emanuele Berrilli, Matteo Garzia

**Affiliations:** 1 Department of Health, Life & Environmental Sciences - University of L’Aquila, Via Vetoio snc, 67100 L’Aquila-Coppito, Italy University of L’Aquila L’Aquila Italy; 2 Ecosystem-Based Management of Marine Resources, Environment and Life Sciences Research Center, Kuwait Institute for Scientific Research, Hamad Al-Mubarak Street, Building 900004, Area 1, Ras Salmiya, Kuwait, Kuwait Kuwait Institute for Scientific Research Kuwait Kuwait; 3 Honorary Research Fellow, National Museum of Wales, Cardiff CF10 3NP, Wales, UK National Museum of Wales Cardiff United Kingdom

## ﻿Introduction

Salvi et al. (2022) introduced the new species *Ostreaoleomargarita* in the on-line-only journal Journal of Zoological Systematics and Evolutionary Research. However, this name was not registered in ZooBank and under ICZN rules (ICZN 2012; Krell and Pape 2015) this name is not currently available. Unfortunately, the Journal of Zoological Systematics and Evolutionary Research was unable to publish a corrigendum in 2022. Therefore, we provide here a Code-compliant description of *Ostreaoleomargarita* Oliver, Salvi, and Al-Kandari, sp. nov. as reported in Salvi et al., (2022) with the ZooBank registration number of the publication. See Salvi et al. (2022) for detailed results of phylogenetic, species delimitation, and morphological analyses on which the systematic assessment of this new species was based. A further emendation to Salvi et al. (2022) concerns the scale bars in Figs 6 and 7 that were wrongly modified during the editorial process but are correctly shown here in Figs 1 and 2.

**Figure 1.**
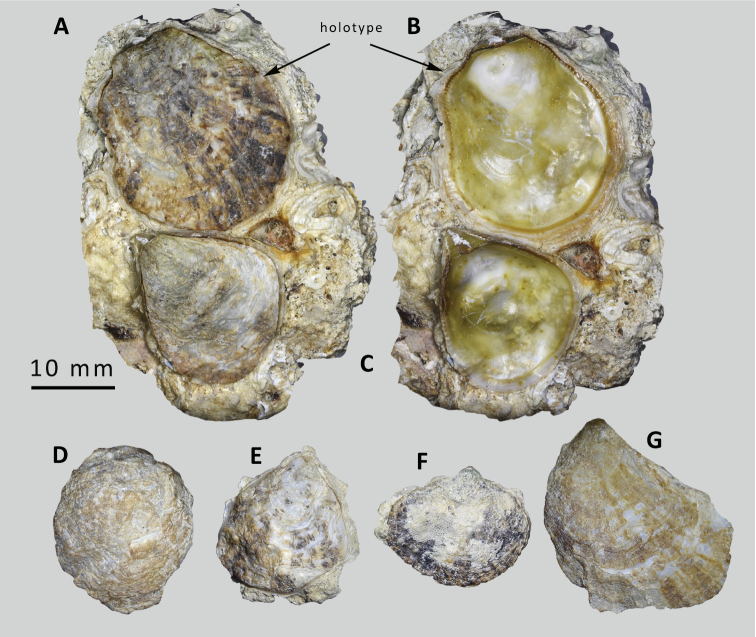
*Ostreaoleomargarita* sp. nov. **A** holotype upper shell in situ with another specimen, Al Sha’ab **B** holotype upper shell, internal view of attached valves showing catachomata, greenish interior and brown margin [NMW.Z.2021.009.008] **C–F** paratypes, variations in external sculpture and colouration, all Al Sha’ab **D** brown, tubercular **E** black and beige, foliar **F** Al Sha’ab site typically encrusted with Bryozoa; [NMW.Z.2021.009.010.] **G** Failaka, rayed, foliar [NMW.Z.2021.009.011 #7].

**Figure 2.**
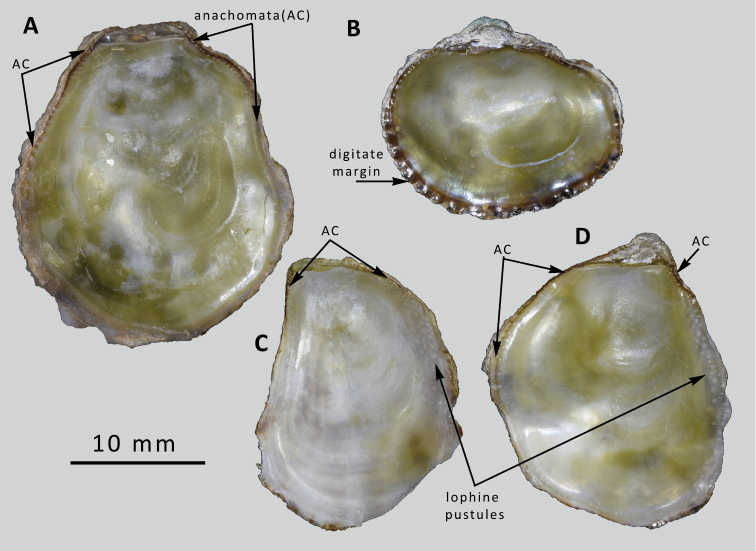
*Ostreaoleomargarita* sp. nov. Internal views of upper valves to show differences in marginal form and chomata **A** holotype, Al Sha’ab, anachomata and smooth ventral margin [NMW.Z.2021.009.008] **B** Al Sha’ab, digitate margin [NMW.Z.2021.009.010] **C** Failaka, weak anachomata and lophine pustules **D** Al Sha’ab, anachomata and lophine pustules [NMW.Z.2021.009.010].

**Nomenclatural acts.** The new name contained in this corrigendum is available under the International Code of Zoological Nomenclature. This work and the nomenclatural act it contains have been registered in ZooBank. The ZooBank Life Science Identifier (LSID) for this publication is urn:lsid:zoobank.org:pub:CE471079-3537-44AF-9CBA-5A070FAFDC1A.

***Ostreaoleomargarita* Oliver, Salvi & Al-Kandari, sp. nov.**

Figs 1, 2

**Type material.** All type material was deposited in the National Museum of Wales NMW.Z. 2021.009.008–.012.

Kuwait • **Holotype**, 1 complete shell attached to rock. Kuwait City, Al Sha’ab, 29.3675°N, 48.0244°E. Low intertidal, attached under rock on gravel. Coll. PG. Oliver, December 2019, not sequenced. NMW.Z.2021.009.008 (Figs 1A, B, 2A).

Kuwait • **Paratypes**, 11 spec. used for sequencing NMW.Z.2021.009.009/#1–#11. 4 figured shells, data as holotype. NMW.Z.2021.009.010/#1–#4. (Figs 1C–F, 2B, 3D).

Kuwait • **Paratypes**, 12 spec. used for sequencing, NMW.Z.2021.009.011/#1–#12; #7 figured in Figs 1G, 2C). Ras Al Liwan, Failaka Island, 29.3902°N, 48.3988°E. Low intertidal, attached under rocks. Coll. PG Oliver, December 2019. 9 shells + 5 upper valves, not sequenced, NMW.Z.2021.009.012., Ras Al Liwan, Failaka Island, 29.3902°N, 48.3988°E. Low intertidal, attached under rocks. Coll. PG Oliver, December 2019.

**Other material examined.** Kuwait • 20 shells attached to rock. Kuwait City, Al Sha’ab, 29.3675°N, 48.0244°E. Low intertidal, attached under rock on gravel. Coll. PG. Oliver, December 2019. NMW.Z.2021.009.013.

**Shell description.** Small shells up to 25 mm in height (beak to ventral margin); dimensions of upper valve of holotype are 20.2 × 17.5 mm. Shell thin but robust. Approximately circular, oval to pyriform.

Lower valve shallowly cupped to irregular; cemented for most or all of its attachment, margin smooth or, if free, then finely plicate. Non-nacreous margin very narrow. Ligament area narrow, elongated in some. Prominent catachomata on both anterior and posterior dorsal margins. Adductor muscle large, reniform. Interior colour flushed olive-green, paler in some smaller shells, most with a distinct narrow brown marginal band.

Upper valve flat, irregular to domed. Anachomata corresponding to catachomata. Sparse elongate tubercles (lophine pustules) on posterior ventral edge in some valves. Internal colour as that of lower valve. Outer surface usually obscured by epifaunal growths, typically Bryozoa, calcareous algae, worm tubes, or other oysters. Sculpture mostly of flattened foliar scales, some areas finely tubercular; colour of radial bands rust brown to black on a grey-beige ground, some almost uniform ground colour. Thin chalky layers present most visible in attached valves.

**Type locality.** Kuwait, Kuwait City, Al Sha’ab, 29.3675°N, 48.0244°E. Intertidal, under rocks.

**Distribution.** Intertidal shores of Kuwait.
